# Psychiatry Trainees’ Wellbeing in the Northwest: Insights from Focus Groups

**DOI:** 10.1192/bjo.2026.11275

**Published:** 2026-06-30

**Authors:** Heidi Soliman, Wala Elabbas, Anais Alexander, Neelo Aslam

**Affiliations:** 1 Lancashire and South Cumbria NHS Foundation Trust, Preston, United Kingdom; 2 Pennine Care NHS Foundation Trust, Manchester, United Kingdom; 3 Manchester Foundation Trust, Manchester, United Kingdom

## Abstract

**Aims:**

Trainees’ wellbeing is constantly becoming an area of increased interest in the recent years. The impact of the pandemic, increased workload while balancing exams, training requirements, and family and caring responsibilities can pose significant demands on trainees. Psychiatry trainees are not immune to those challenges, but furthermore, they are susceptible to challenges unique to psychiatry – such as patient suicide, working in a field that still suffers from stigma from both patients and healthcare professionals. Despite the presence of resources, wellbeing remains an area for improvement.

In our study, we sought to explore areas that impact the wellbeing of psychiatry trainees in the northwest of England; the challenges encountered and the factors that contributed both negatively and positively to health and wellbeing.

**Methods:**

We conducted online focus groups with psychiatry trainees in different stages in their training (CT1-ST8), to explore factors affecting health and wellbeing. Each session was co-facilitated by the research team, and structured into three sections: challenges faced, unhelpful experiences, and helpful experiences. Sessions were transcribed via Microsoft Teams, with participants informed of confidentiality and anonymity, and demographic data collected via an anonymous online questionnaire. Transcripts were thematically analysed by the research team to identify key themes.

**Results:**

Trainees reported multiple factors negatively affecting their wellbeing, including the rotational nature of the job, significant workload, lack of rest facilities, lack of appropriate supervision, and lack of individualised support for trainees with personal vulnerabilities. Challenges were compounded by difficulties navigating administrative systems and accessing occupational health or professional support. Helpful factors included supportive and flexible supervisors, strong team relationships, good workplace integration, as well as respecting and nurturing trainees’ autonomy and sharing constructive feedback.

**Conclusion:**

The quality of the trainee–supervisor relationship emerged as the most important factor influencing wellbeing, with emotionally intelligent supervisors who notice struggles and encourage reflective practice being particularly beneficial. While multiple support resources exist, they are often not tailored to individual needs or widely known; targeted training for supervisors, including guidance on supporting neurodiverse trainees, may improve accessibility and effectiveness of wellbeing support.

